# Disparities in Prevalence and Barriers to Hypertension Control: A Systematic Review

**DOI:** 10.3390/ijerph192114571

**Published:** 2022-11-06

**Authors:** Mohamed Hassan Elnaem, Manar Mosaad, Doaa H Abdelaziz, Noha O. Mansour, Abubakar Usman, Mahmoud E. Elrggal, Ejaz Cheema

**Affiliations:** 1 Department of Pharmacy Practice, Faculty of Pharmacy, International Islamic University Malaysia, Kuantan 25200, Malaysia; 2 Quality Use of Medicines Research Group, Faculty of Pharmacy, International Islamic University Malaysia, Kuantan 25200, Malaysia; 3 Department of Internal Medicine, Ministry of Health, Alexandria Governorate 5517176, Egypt; 4 Pharmacy Practice & Clinical Pharmacy Department, Faculty of Pharmacy, Future University in Egypt, Cairo 4740011, Egypt; 5 Department of Clinical Pharmacy and Pharmacy Practice, Faculty of Pharmacy, Mansoura University, Mansoura 7650030, Egypt; 6 Discipline of Clinical Pharmacy, Universiti Sains Malaysia, Penang 11800, Malaysia; 7 College of Pharmacy, Umm Al-Qura University, Makkah 21955, Saudi Arabia; 8 School of Pharmacy, University of Management and Technology, Lahore 54770, Pakistan

**Keywords:** hypertension, blood pressure, prevalence, barriers, medication adherence, awareness, antihypertensive agents

## Abstract

Controlling hypertension (HTN) remains a challenge, as it is affected by various factors in different settings. This study aimed to describe the disparities in the prevalence and barriers to hypertension control across countries of various income categories. Three scholarly databases—ScienceDirect, PubMed, and Google Scholar—were systematically examined using predefined search terms to identify potentially relevant studies. Original research articles published in English between 2011 and 2022 that reported the prevalence and barriers to HTN control were included. A total of 33 studies were included in this systematic review. Twenty-three studies were conducted in low and middle-income countries (LMIC), and ten studies were from high-income countries (HIC). The prevalence of hypertension control in the LMIC and HIC studies ranged from (3.8% to 50.4%) to (36.3% to 69.6%), respectively. Concerning barriers to hypertension control, patient-related barriers were the most frequently reported (*n* = 20), followed by medication adherence barriers (*n* = 10), lifestyle-related barriers (*n* = 8), barriers related to the affordability and accessibility of care (*n* = 8), awareness-related barriers (*n* = 7), and, finally, barriers related to prescribed pharmacotherapy (*n* = 6). A combination of more than one category of barriers was frequently encountered, with 59 barriers reported overall across the 33 studies. This work reported disparities in hypertension control and barriers across studies conducted in LMIC and HIC. Recognizing the multifactorial nature of the barriers to hypertension control, particularly in LMIC, is crucial in designing and implementing customized interventions.

## 1. Introduction

Persistent hypertension (HTN), or high blood pressure (BP), is a significant public health concern, with an estimated affected population of nearly 1.3 billion people worldwide [1]. It is considered a global epidemic disease that imposes a considerable health and economic burden on the healthcare system across low- and middle-income countries (LMIC) and high-income countries (HIC) [2]. Hypertension is a major factor contributing to the increased CVD mortality rate and is one of the major causes of premature mortality [3].

Hypertension treatments should keep blood pressure under optimal control to avoid negative consequences. Evidence has shown that patients with an excessive morning rise in BP and those who lack a nocturnal decrease in BP have an increased incidence of strokes, heart failure, and other CV events [4]. However, HTN control remains suboptimal; it can be achieved in less than half of hypertensive patients even though more patients have received treatment over time [3]. The inability to achieve and maintain HTN control is a significant healthcare concern, wherein patients with untreated or inadequately controlled HTN have a significantly increased risk of disease complications [1].

Hypertension prevalence, awareness, and control are subject to several disparities globally [5]. For example, the relative decrease in the age-standardized prevalence of hypertension by 2.6% in HIC was associated with a relative increase in the awareness, treatment, and control proportions. Meanwhile, in LMIC, the decrease in awareness and control has contributed to a rise of 7.7% in hypertension prevalence [5]. These observations indicate the need for coordinated BP control interventions considering awareness, optimal treatment, and long-term control.

Identifying significant barriers that may prevent patients from attaining HTN control is critical. Additionally, there is a need for recent data on comparative prevalence values and the patient-related barriers hindering optimal BP control across LMIC and HIC. Although there are recent data from population-based studies covering hypertension prevalence, control, and treatment [5], patient-reported barriers to attaining HTN control have not been extensively and systematically presented in the recent literature. Therefore, this study was conducted to describe the disparities in the prevalence of and barriers to HTN control and to outline implications for customized hypertension care across various income categories.

## 2. Methods

The Preferred Reporting Items for Systematic Reviews and Meta-Analyses (PRISMA) guidelines were employed to report the findings of this systematic review [6].

### 2.1. Data Sources and Search Strategy

**Figure 1:**
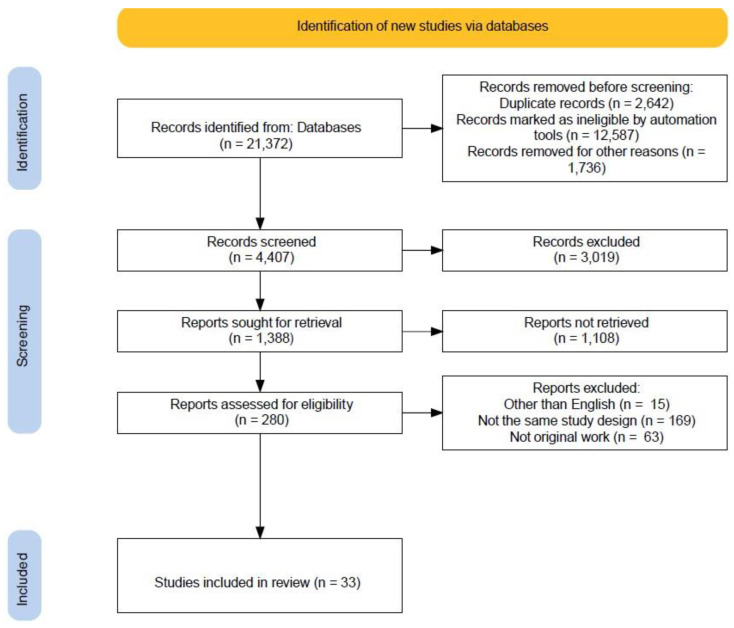
PRISMA Flowchart of the selection process of the reviewed studies.

Three scholarly databases—ScienceDirect, PubMed, and Google Scholar—were thoroughly and systematically examined. The search terms “prevalence AND barriers OR factors OR challenges AND blood pressure control OR hypertension control” were used interchangeably across the databases to identify potentially relevant studies. Additional search of the databases with the keywords combined with individual country names was performed to ensure the inclusion of all relevant articles that might not be retrieved in the non-country-specific initial search. The original research articles published between January 2011 and February 2022 reported the prevalence of hypertension control and provided insight into the potential determinants of hypertension control. The rationale behind restricting the searches to eleven years was to provide the most recent insight into the research topic from patients’ perspective. The flowchart describing the selection of the studies is presented in Figure 1.

### 2.2. Study Screening and Selection

We included original research articles published between January 2011 and February 2022 that used a cross-sectional design and survey methodology. The inclusion criteria were the reporting of the prevalence of hypertension control and its possible determinants. Studies that combined the prevalence of hypertension, its treatment, and its control were included, but we only reported the figures on control among hypertensive patients, not in all study populations. Two independent reviewers, MHE and MM, scrutinized the titles and abstracts of the studies. Then, potentially relevant studies were further reviewed using predefined inclusion criteria of study design, methodology, and publication year. The findings of the two reviewers regarding the study selection were compiled and compared. Disagreements were resolved through the involvement of a third reviewer. The two reviewers convened a series of meetings to determine which studies included all the details required for data extraction to maintain consistency. The full text of eligible studies was retrieved for inclusion and methodological quality assessment.

### 2.3. Data Extraction

The data for the selected studies were extracted using a predesigned data extraction form that included the primary author’s name, country, study design, objectives, key findings, and conclusions. Additionally, the included studies were classified according to their country’s economic status provided by the World Bank’s country classification system according to income. The classification of studies into those from LMIC and HIC was performed only after the final decision regarding the included studies.

### 2.4. Eligibility Criteria

Cross-sectional studies were included, including patient surveys that reported prevalence and patient-reported barriers to hypertension control. The exclusion criteria included studies that used experimental designs to improve blood pressure control but did not report the associated barriers. Additionally, reviews, commentaries, protocol-based studies, book chapters, conference proceedings, and meta-analysis studies were excluded.

### 2.5. Quality Assessment

The methodological quality appraisal of the included studies was performed using the risk of bias instrument for cross-sectional surveys contributed by the CLARITY Group [7]. Each quality item was graded on a scale of four choices: definitely yes (low risk of bias), probably yes, probably no, and definitely no (high risk of bias). The items used to evaluate bias in each trial included the sample representation, the response rate, missing data, clinical sensibility, validity, and reliability of the surveys. Supplementary Table S1 shows the quality assessment of the included studies.

### 2.6. Barriers to Hypertension Control

For a consistent and systematic comparison, we categorized all reported barriers in the included studies into one of six main categories: patient-related barriers (demographic and comorbidity), awareness-related barriers (knowledge and health literacy), lifestyle-related barriers (dietary habits and physical inactivity), affordability and accessibility of care, medication adherence, and pharmacotherapy-related barriers (duration and number of prescribed medicines).

## 3. Results

### 3.1. Overview of the Included Studies

Thirty-three studies were eligible for inclusion in this review. Two studies were conducted in low-income countries, namely, Ethiopia [8] and Rwanda [9]. Eleven studies were conducted in lower-middle-income countries, which are Angola [10], Tanzania [11], Senegal [12], Cameron [13], Nigeria [14,15], Ghana [16,17], Nepal [18,19], and Vietnam [20]. Whereas ten studies were undertaken in upper-middle-income countries, comprising China [21,22,23,24,25,26] and one study each for Brazil [27], Botswana [28], Iraq [29], and Peru [30]. The remaining ten studies were conducted in high-income countries (HIC), which are Australia [31], Sweden [32], Hong Kong [33], Saudi Arabia [34], Chile [35], South Korea [36], Germany [37], Spain [38] Ireland [39], and Singapore [40]. For another way of viewing the included studies, fourteen were from Asian countries, whereas eleven primarily represented African countries, four were from European countries, three were from South America, and one was from the western pacific region. This regional country distribution might indicate how the burden and challenges with optimal HTN management differ globally.

The data across different countries varied significantly as they were affected by the population size. Therefore, in this section, we report only the lower and higher prevalence values reported across countries’ economic groups. Thus, the prevalence of hypertension control in the LMIC and HIC studies ranged from (3.8% to 50.4%) and (36.3% to 69.6%), respectively. Overall, higher percentages of hypertension control were reported in HIC compared to other countries. Table 1 summarizes all the included studies and their relevant characteristics.

### 3.2. Methodological Quality of Included Studies

The quality of all included studies was appraised using the five criteria determined by the assessment tool. Overall, no study has fulfilled a low risk of bias across the five categories. Several studies have achieved low bias risk in the items related to the study population and response rate. However, most studies had a potentially high bias risk in the items related to the reported reliability and validity of the used questionnaire. This point should be carefully considered in future cross-sectional surveys reporting clinical measures. Therefore, maintaining consistency in the detailed reporting of the tools’ reliability and validity is critical. A summary of the quality appraisal of the included studies is provided in supplementary Table S1.

### 3.3. Barriers to Hypertension Control

All barriers reported across all the studies were classified under these six main categories: patient-related barriers (demographic and comorbidity), awareness-related barriers (knowledge and health literacy), lifestyle-related barriers (dietary habits and physical inactivity), affordability and accessibility of care, medication adherence, and pharmacotherapy-related barriers (duration and number of prescribed medicines). Examples of the barriers under these six main categories are provided in Table 2.

Across all included studies, patient-related barriers were the most frequently reported barriers to hypertension control (*n* = 20), followed by medication adherence barriers (*n* = 10), lifestyle-related barriers (*n* = 8), barriers related to affordability and accessibility of care (*n* = 8), awareness-related barriers (*n* = 7), and, finally, barriers related to the prescribed pharmacotherapy (*n* = 6). The number beside each category refers to the frequency of reporting across the included 33 studies. In addition, from the reported numbers, it is evident that a combination of more than one category of barriers was frequently encountered, with 59 barriers reported overall across the 33 studies. Studies from LMIC more frequently reported a variety of barriers. In contrast, two-thirds of the barriers in the HIC studies belong to the patient-related barrier category, most commonly that of advancing age. Table 3 shows the frequency of the reported barriers per category across all countries classified by income.

## 4. Discussion

This work reviewed the BP control prevalence and patient-related barriers in 33 studies conducted in various LMICs (*n* = 23) and HIC (*n* = 10). We have observed several disparities in the prevalence of hypertension control across different countries owing to differences in the population size and the encountered barriers. Overall, the patient-related barriers were the most frequently reported barriers to hypertension control (*n* = 20), followed by medication adherence barriers (*n* = 10), lifestyle-related barriers (*n* = 8), barriers related to the affordability and accessibility of care (*n* = 8), awareness-related barriers (*n* = 7), and, finally, barriers related to prescribed pharmacotherapy (*n* = 6). The studies from LMIC more frequently reported a combination of barriers that covers all six barrier categories, while two-thirds of the barriers in the HIC studies belong to the patient-related-barrier category, most commonly that of advancing age. This study has classified and summarized the identified barriers and has provided indications for improving hypertension control.

### 4.1. Barriers to Optimal Hypertension Control

#### 4.1.1. Patient-Related Barriers

Across all the included studies, these barriers were the most frequently reported, with a relatively higher impact on hypertension control in the HIC studies. Two main subcategories were identified, which include sociodemographic factors and comorbidities.

##### Sociodemographic Factors

Sociodemographic characteristics, including age, gender, and socioeconomic status, were reported to impact BP control. For example, an Iraqi study demonstrated that non-elderly male patients were more likely to have uncontrolled HTN regardless of their education level and employment status [29]. This might be explained by the difference in the healthcare-seeking behavior of non-elderly compared to elderly patients who had potentially more opportunities to seek healthcare services triggered by their underlying comorbidities and their need for regular follow-up visits. However, some studies in HIC highlighted that old age is a barrier to optimal BP control and a strong predictor of uncontrolled HTN [40,41]. Potential explanations include changes via physiological aging, comorbidities, the heterogeneity of the treatment among elderly patients, social exclusion, a lack of support, and insufficient knowledge about HTN self-management [40].

Concerning the gender impact on BP control, several studies in Chile, Singapore, and Tanzania highlighted that male patients were more prone to having poor BP control [11,35,40]. Meanwhile, Santosa et al. reported higher odds of BP control among male patients with diabetes in Sweden [32]. The gender-based differences in hypertension control could be attributed to differences in the patients’ levels of concern and awareness of their hypertension. For example, a recent German study highlighted that women tend to be more concerned about their BP control than men [37]. Additionally, women tended to be more aware of their hypertension than men, as reported in two studies in Angola and Peru [10,30]. Men and premenopausal women of similar ages are at greater risk of CVD and renal diseases. However, BP increases to higher levels in postmenopausal women than in men [42]. This shows that gender barriers to optimal BP control depend on other risk factors such as age and health conditions such as menopause. It has also been suggested that BP levels in both genders are more likely to increase as the number of risk factors increases [22].

Furthermore, in a study conducted in LMIC, there was no significant association between education, employment level, and BP control [29]. In contrast, the HIC study’s findings showed that patients with less than a high school education were more likely to be resistant to HTN treatment [43]. In addition, it was reported that a higher rate of uncontrolled BP is observed among people with low education levels [44].

Unsurprisingly, the LMIC studies tended to highlight the role of financial constraints and their adverse impact on medication adherence, leading to the suboptimal achievement of BP control [45]. Overall, socioeconomically disadvantaged people are more likely to have HTN and have a higher likelihood of having poor BP levels despite treatment [44]. This might underpin the need for customized interventions to target BP control improvement among individuals affected by socioeconomic challenges. Moreover, recent research has highlighted the impact of sociodemographic factors on hypertension control in the era of the COVID-19 pandemic. The findings showed that the pandemic negatively impacted the overall hypertension control of the younger population, the lower-income group, unmarried, and unemployed individuals [46].

##### Comorbidity

Comorbidity has various implications, including the necessity of a more complex therapeutic regimen and the increased likelihood of poor adherence to antihypertensives [47]. However, it can be argued that the presence of a comorbidity results in more chances of seeking healthcare services and, consequently, having higher odds of controlling risk factors [45]. Several studies, mainly in LMIC, have demonstrated a strong association between diabetes mellitus (DM) and poor BP control [27,29]. Besides poor BP control resulting from DM as a comorbidity, HTN plays a significant role in DM progression. In type 2 DM, HTN is often present in the insulin resistance syndrome in obesity and dyslipidemia. Meanwhile, in type 1 DM, HTN may indicate the onset of diabetic nephropathy [48]. The coexistence of HTN and DM necessitates a multifaceted approach considering the target levels and appropriate therapy selection [49,50].

Furthermore, across the LMIC and HIC studies, inadequate BP control was reported among those with elevated total cholesterol, LDL, and uric acid levels [23,31,38]. Additionally, stable angina pectoris significantly interfered with adequate BP control among hypertensive patients [23]. In addition, a strong association was demonstrated between CKD and poor BP control, particularly in the case of severe albuminuria or proteinuria [31]. Moreover, depression may interfere with BP control, with a significant correlation between systolic and diastolic BP levels and depression [51].

#### 4.1.2. Medication Nonadherence

Suboptimal medication adherence was more pronounced in the LMIC studies (*n* = 9). A total of five studies have reported that more than 40% of their study participants had poor adherence to their antihypertensive medications [14,15,16,21,27]. In addition, the evidence shows that poor BP control is significantly associated with poor adherence [16,21]. Two Nigerian studies reported that forgetfulness was the most common reason for poor adherence, followed by financial barriers, a high pill burden, and the side effects of the antihypertensive medications [14,15]. Another study highlighted that a young age, living in rural areas, the fear of becoming accustomed to the medication, and unsatisfactory treatment are common barriers to optimal adherence [52]. A recent study reported that patients who omitted their prescribed antihypertensive medications showed more inadequate BP control [28].

Nonetheless, additional evidence suggests that polypharmacy substantially affects blood pressure control more than medication adherence alone [53]. These findings show that medication adherence and BP control are multifactorial, implying the need for collaborative, multifaceted interventions for positive clinical outcomes [54].

#### 4.1.3. Lifestyle Factors

This study has frequently identified lifestyle-related BP control barriers in several LMIC and HIC studies. Approximately 60% of the factors related to individual health and quality of life are linked to lifestyle, indicating that those with unhealthy lifestyles are more likely to encounter morbidity, disability, and mortality [55]. The association between lifestyle factors, including physical inactivity, smoking, BMI, salt intake, alcohol consumption, stress, and poor BP control, has been confirmed [22,36,38,41]. Patients who perform physical activity at least four days per week are more inclined to have optimal BP control [36]. Moreover, smoking and an abnormally high BMI are independent predictors of elevated systolic BP [41]. It is worth highlighting that the parameters used to measure lifestyle factors vary among the reviewed studies. In addition, several factors could coexist in one patient. Therefore, a Chinese study that examined the impact of lifestyle factors on BP control highlighted that BP control was positively correlated with the number of lifestyle factors being addressed [22].

#### 4.1.4. Pharmacotherapy-Related Barriers

The challenge of optimizing HTN pharmacotherapy has been highlighted exclusively across numerous LMIC studies. Monotherapy is recommended in those whose blood pressure is less than 20/10 mm Hg above the target; however, combination therapy would benefit those with a BP more than 20/10 mm Hg above the target [56]. The failure to intensify and optimize the pharmacotherapy with disease progression is significantly associated with lower odds of BP control [57]. However, uncontrolled hypertension was reported among patients on two or more antihypertensives, indicating a mixed relationship between the number of antihypertensives and BP control [41,43]. In a recent scientific statement by the American Heart Association, complexity, frequent changes, and a lack of immediate benefit of the treatment regimen are common contributors to nonadherence [58]. All of these therapy-related factors are becoming more frequent with the increasing the number of antihypertensives.

Besides being offered monotherapy or combination therapy, the choice of antihypertensive agent affects BP control. According to a Chinese study that explored the BP control barriers among patients with CVD, a correlation between non-dihydropyridine CCB and a lower rate of BP control was confirmed [23]. However, the extent of BP reduction should remain the primary determinant of reducing CVD risk among hypertensive patients, wherein the role of combination therapy is increasingly needed [56].

Moreover, long-term antihypertensive treatment is reported as a facilitator of medication adherence [59]. In addition, a longer duration of taking antihypertensive drugs is positively associated with adequate BP control [8]. The avoidance of medication errors is essential for optimizing HTN pharmacotherapy, considering that medication errors represent a significant predictor of uncontrolled HTN [28]. Overall, as pharmacotherapy’s optimization is not a one-time intervention in people with chronic diseases, continuous assessments of the effectiveness and safety alongside interventions to boost adherence are increasingly needed to improve clinical outcomes [54,60]. Further detailed evaluations of the ongoing prescribing patterns will provide a basis for interventions that are most likely to be adopted in clinical practice [61,62].

### 4.2. Implications for Hypertension Control across Different Countries

The BP control barriers’ distribution appears differently due to the older ages in HIC and inadequate knowledge, poor medication adherence, and challenges regarding pharmacotherapy optimization in LMIC. Furthermore, the HIC studies have reported that older age is a determinant of suboptimal BP control [31,63]. These findings imply the need for more coordinated hypertension care for the older population with fewer caregivers and regular follow-ups and medication optimization support. It is also an area of further need for patient safety initiatives to support better hypertension control in this patient population.

Interventions to improve disease knowledge and enhance medication adherence are required, particularly in LMIC, to help hypertensive patients cope with long-term disease control [54]. The knowledge gaps among patients with chronic diseases are common and need to be addressed by relevant educational interventions [64]. In a systematic review of nonadherence to antihypertensive medications in LMIC, the findings suggested that medication adherence ranged from 25.4% to 63.4%, depending on the cut-off point scales (80–90%) or the MMAS eight-item scale, respectively [65]. Apart from these relatively special considerations, there is a generic need for community interventions to enforce a healthier lifestyle and pharmacotherapy optimization in most LMIC studies. Interventions that optimize pharmacotherapy which consider the simplifications of drug regimens are needed [66].

Finally, an examination of the frequency of the barriers encountered in LMIC studies indicates the need for interventions that tackle several barriers simultaneously. There is a need for the simultaneous consideration of barriers related to lifestyle, disease knowledge, medication adherence, pharmacotherapy optimization, and the cost of healthcare services. The evidence shows that multifaceted interventions that analyze all potential barriers and target them in a stepwise customized approach would be associated with significant BP control improvement in the community [67,68]. Care for chronic diseases, including hypertension, requires coordinated interprofessional care that involves multiple health industry team members. Collaborative models involving physicians, pharmacists, and nurses are feasible and promising with respect to proper design and coverage for all BP control determinants [69,70].

This study is not without limitations. Although this work provides a recent and comprehensive insight into the prevalence and patient-related barriers to BP control generated from survey-based studies in the last decade, it did not account for the considerable difference in the study populations between studies. All the limitations of survey studies are the inherent limitations of this review, such as reporting and social desirability biases. Finally, this work could be restricted to a systematic description of the findings due to the lack of planned statistical inferences.

## 5. Conclusions

This work identified disparities in the types and frequencies of barriers to hypertension control across LMIC and HIC studies. Although patient-related barriers were common in both groups, they were relatively higher in HIC studies. The barriers to medication adherence, pharmacotherapy optimization, disease knowledge, and lifestyle must be addressed by multifaceted interventions in developing countries. Recognizing the multifactorial nature of the barriers to hypertension control, particularly in LMIC, is crucial for designing and implementing customized interventions.

## Figures and Tables

**Table 1:** Summary of the included studies and their relevant characteristics (*n* = 33).

No	Author	Country and Income Class	Sample Included	Significant Findings
1	Animut, et al., 2018 [8]	Ethiopia (Low-income)	395 hypertensive patients.	50.4% had their BP controlled. Salt intake, overweight, and obesity were negatively associated with BP control. Physical activity, duration on antihypertensive drugs (2–4 or ≥5 yrs.), and high adherence were positively correlated with BP control.
2	Sibomana, et al., 2019 [9]	Rwanda (Low-income)	112 patients from four district rural hospitals.	29% had their BP controlled. 77% reported medication adherence associated with literacy and lack of adverse effects. 50% highlighted physicians’ nonadherence to clinical guidelines.
3	Paquissi, et al., 2016 [10]	Angola (Lower-middle-income)	102 hypertensive patients.	7.8% had their BP controlled, while 54.9% and 28.4% were aware and treated, respectively. Younger (<37 years) and male patients were more likely to be unaware of their disease.
4	Duboz, et al., 2014 [12]	Senegal (Lower-middle-income)	165 hypertensive patients.	5.4% had their BP controlled, while 27.8% and 17% were aware and treated, respectively. Older patients (≥50 years) were more likely to be aware of and treated for their HTN.
5	Zack, et al., 2016 [11]	Tanzania (Lower-middle-income)	803 hypertensive patients.	10% had their BP controlled, while 48% and 22% were aware and treated, respectively. Higher BP readings were reported in male, older, uneducated, unemployed, overweight, obese, and physically-inactive patients.
6	Menanga, et al., 2016 [13]	Cameroon (Lower-middle-income)	440 hypertensive patients in an urban city.	36.8% had their BP controlled. Optimal medication adherence and dietary lifestyle changes were significantly associated with BP control.
7	Okwuonu, et al., 2014 [14]	Nigeria (Lower-middle-income)	252 adults with hypertension.	32.9% had their BP controlled (affected by knowledge and lifestyle changes) Low medication adherence was reported in 68.7% of patients due to forgetfulness (61.2%), financial barriers (56.6%), a heavy pill burden (22.5%), and side effects (17.3%).
8	IIoh, et al., 2013 [15]	Nigeria (Lower-middle-income)	140 adults with primary HTN on treatment for at least six months.	35% had their BP controlled, and 42.9% were adherent. Adherence, HTN duration (≥3 years), and receiving > one anti-HTN therapy were related to better blood pressure control.
9	Sarfo, et al., 2018 [16]	Ghana (Lower-middle-income)	2870 hypertensive participants enrolled at five different hospitals.	42.3% had their BP controlled. Uncontrolled BP was attributed to receiving therapy at a tertiary care level, longer HTN duration, poor adherence, and number of and access to anti-HTN treatments.
10	Harrison, et al., 2021 [17]	Ghana (Lower-middle-income)	310 hypertensive participants	41.8% had their BP controlled. Affordability (OR, 1.917; 95% CI: 1.013–3.630) and accessibility (OR, 1.642; 95% CI, 0.843–3.201) were more significantly linked to blood pressure control.
11	Gala, et al., 2020 [28]	Botswana (Upper-middle-income)	280 adult patients with HTN on medications.	45% had their BP controlled. 34% had ≥ one medication error. Having ≥ one medication error was significantly associated with uncontrolled HTN compared with no errors.
12	Devkota, et al., 2016 [18]	Nepal (Lower-middle-income)	191 hypertensive patients.	24% had their BP controlled, while 61.8% and 78.8% were aware and treated, respectively. BP control was associated with combination therapy, medication adherence, follow-up care, and healthcare providers’ counselling
13	Dhungana, et al., 2022 [19]	Nepal (Lower-middle-income)	2792 hypertensive patients.	Only 3.8% had their BP controlled. About 10.3% received antihypertensive treatment. 20% were aware of their hypertension.
14	Son, et al., 2012 [20]	Vietnam (Lower-middle-income)	2467 hypertensive patients.	Only 10.7% had their BP controlled. About 29.6% received antihypertensive treatment. 48.4% were aware of their hypertension.
15	De Souza, et al., 2014 [27]	Brazil (Upper-middle-income)	383 adult patients with HTN.	33.7% had their BP controlled. Only 54.3% reported adherence to anti-HTN therapy. Diabetes mellitus (DM) was observed in 31% of participants, with only 15.7% having their BP controlled.
16	Lerner, et al., 2013 [30]	Peru (Upper-middle-income)	205 hypertensive patients.	4.9% had their BP controlled, while 48.3% and 40% were aware and treated, respectively. Women were more aware of their HTN than men.
17	Nassr, et al., 2019 [29]	Iraq (Upper-middle-income)	300 adult patients with hypertension.	38.7% had their BP controlled. Age < 60 years, male gender, and diabetes were predictors of uncontrolled BP.
18	Wang, et al., 2013 [21]	China (Upper-middle-income)	556 hypertensive patients from a rural community.	12.5% had their BP controlled among only 429 patients aware of being hypertensive. Optimal HTN control was hindered by inadequate knowledge (82.8%), treatment cost (39.4%), poor medication adherence (65%), and lack of counselling sessions (95.1%).
19	Li, et al., 2016 [22]	China (Upper-middle-income)	31,694 adult respondents were diagnosed with HTN.	29.5% had their BP controlled. Higher BP levels positively correlate with the number of risk factors in both genders.
20	Xu, et al., 2013 [23]	China (Upper-middle-income)	3279 HTN and CHD patients.	18% had their BP controlled. Non-dihydropyridine CCB was associated with a low BP control rate. Independent factors of poor BP control include being overweight, stable angina pectoris, and a family history of diabetes.
21	Chen, et al., 2020 [24]	China (Upper-middle-income)	89,925 hypertensive patients.	25.4% had their BP controlled. Lower odds of uncontrolled BP were reported in women, those with diabetes, and CHD. Older patients, current smokers, and monotherapy users had higher odds of uncontrolled BP.
22	Lei wu, et al., 2015 [25]	China (Upper-middle-income)	1409 elderly (≥60 years) with hypertension.	30.3% had their BP controlled, while 74.5% and 63.7% were aware and treated, respectively. BP control was significantly associated with higher education levels, family history of HTN, and CVD comorbidity.
23	Xia, et al., 2021 [26]	China (Upper-middle-income)	1046 hypertensive patients.	48.3% and 37.6% had their BP controlled in public vs. private clinics. Higher treatment (87.5% vs. 66.8%), higher adherence (91.5% vs. 82.5%), and lower depression levels (8.5% vs. 18.2%) in public vs. private clinics.
24	Santosa, et al., 2020 [32]	Sweden and China (High-income “Sweden”)	Sweden (*n* = 25,511) and China (*n* = 25,356).	47.6% of males and 58.7% of females had their BP controlled in Sweden vs. 33.2% and 37.6% in China. Awareness was higher among patients in Sweden (63.7%” males” and 69.1% “females”) compared to China (50.2%” males” and 44.3% “females”). Higher odds of BP control in Sweden were reported for those with normal weight, controlled lipid profiles, and men with diabetes.
25	Ting li, et al., 2016 [33]	Hong Kong (High-income)	2445 hypertensive patients.	51.3% had their BP controlled, 53.4% had good adherence, and 47.4% had multiple comorbidities. Poor BP control was more likely among those with multiple comorbidities (Diabetes was the most prevalent).
26	Liew, et al., 2019 [40]	Singapore (High-income)	10 215 participants from a multi-ethnic cohort.	37.6% had their BP controlled. Older age was associated with uncontrolled HTN. Younger age, male gender, and lower educational level were associated with untreated HTN.
27	Ham, et al., 2011 [36]	South Korea (High-income)	690 adult patients with HTN on medications.	54.3% had their BP controlled. Higher control rates were observed at a younger age, for those with ≥one comorbidity, and ≥4 days physically active. Being overweight, heavy alcohol consumption, and mild to severe stress reduced BP control.
28	Khayyat, et al., 2017 [34]	Saudi Arabia (High-income)	204 hypertensive patients.	69.6% had their BP controlled. Higher odds were observed with high medication adherence and normal-weight individuals. 46% were adherent. Higher odds were observed among males, older individuals (>65 yrs), and patients with diabetes.
29	Sandoval, et al., 2012 [35]	Chile (High-income)	1194 hypertensive patients.	59.7% had their BP controlled. Women and non-diabetics had better BP control than men and diabetics
30	Zhang, et al., 2019 [31]	Australia (High-income)	1750 CKD patients with HTN.	36.3% had their BP controlled. Those with CVD had lower odds of uncontrolled BP. Participants ≥65 years old and those with severe albuminuria or proteinuria were at higher odds of uncontrolled BP.
31	Murphy, et al., 2016 [39]	Ireland (High-income)	3579 hypertensive adults aged over 50 years.	54.5% and 58.9% were aware and treated (affected by financial barriers). Among those treated, 51.6% had their BP controlled. Higher odds of BP control were observed among those with previous CVD history and those living in rural areas compared with a country area.
32	Tiffe, et al., 2019 [37]	Germany (High-income)	293 adult patients with HTN on medications.	50.2% had their BP controlled. Women who reported higher levels of concern had a higher chance of controlling HTN.
33	Cordero, et al., 2011 [38]	Spain (High-income)	10743 patients with HTN.	55.4% had their BP controlled. BP control rate was similar in those with and without CVD. Higher rates of poor BP control were reported for males, active smokers, obese individuals, and diabetics.

**Table 2:** Overview of common barriers to optimal hypertension control and its examples.

N	Barriers to Hypertension Control		Examples of Individual Barriers
1	Patient-related	Sociodemographic factors	Age: Older age is a strong predictor of uncontrolled HTN
			Gender: The role of gender depends on age and health conditions, such as menopause.
			Socioeconomic status: Socioeconomically underprivileged patients are more prone to suboptimal BP levels despite treatment
			Geographical area: Patients residing in rural areas are more prone to having poor BP control
		Comorbidities	Diabetes: One of the strong predictors of uncontrolled HTN
			Coronary artery disease
			Chronic kidney disease: Patients with severe albuminuria or proteinuria are at greater risk
			Depression: This shows that mental health can also influence BP control
			Hyperlipidemia
			Hyperuricaemia
2	Medication nonadherence		Forgetfulness: The most reported reason
			Financial barrier
			High pill burden
			Side effects of antihypertensive agents
			Low measured BP
3	Lifestyle-related		Smoking
			Obesity
			Salt intake
			Alcohol intake
			Stress
			Physical inactivity
4	Affordability and accessibility-related barriers		Direct cost of treatment
			Cost associated with access to care
			Cost associated with regular follow-up
5	Awareness-related barriers		Disease-related knowledge
			Health literacy regarding risk factors
6	Pharmacotherapy-related		Number of used antihypertensive agents
			Choice of antihypertensive agents
			Duration of taking antihypertensive agents
			Medication error avoidance

**Table 3:** Summary of the categories of hypertension control barriers across countries’ income groups and their overall ranking.

Barriers to Hypertension Control	LMIC Studies (N = 23)	HIC Studies (N = 10)	Total
**Patient-related barriers**	10	10	20
**Medication adherence barriers**	9	1	10
**Lifestyle-related barriers**	6	2	8
**Affordability and accessibility-related barriers**	7	1	8
**Awareness-related barriers**	6	1	7
**Pharmacotherapy-related barriers**	6	0	6
**Total no. of barriers**	44	15	59

## Data Availability

Data are available from the corresponding author upon request.

## Supplementary Materials

The following supporting information can be downloaded at: https://www.mdpi.com/article/10.3390/ijerph192114571/s1, Supplementary Table S1. Quality appraisal of the included studies using the risk of bias instrument for Cross-Sectional Surveys Contributed by the CLARITY Group.
