# Supplementary material for: Disparities in Prevalence and Barriers to Hypertension Control: A Systematic Review
Source: Int J Environ Res Public Health. 2022 Nov 6;19(21):14571. doi: 10.3390/ijerph192114571 (PMC9655663; doi:10.3390/ijerph192114571)
Supplement: Supplementary file 1 [file ijerph-19-14571-s001.zip › ijerph-1906868-supplementary.pdf]

Supplementary Table S1. Quality appraisal of the included studies using the risk of bias instrument for Cross-Sectional Surveys Contributed by the CLARITY Group.

Author & Country	1. Is the source population representative of the population of interest?	2. Is the response rate adequate?	3. Is there little missing data?	4. Is the survey clinically sensible?	5. Is there any evidence for the reliability and validity of the survey instrument?
Animut et al. 2018 [22] Ethiopia	Probably yes	Probably yes	Probably no,	Definitely yes	Probably yes,
Sibomana et al. 2019 Rwanada	Probably no	Probably yes	Probably no	Probably yes	Probably no
Wang et al. 2013 [12] China	Probably yes	Probably yes	Probably no	Definitely no	Definitely no
Li et al. 2016 [13] China	Definitely yes (low risk of bias),	Definitely yes (low risk of bias),	Probably no,	Probably yes,	Probably yes
Xu et al. 2013 [14] China	Definitely yes	Definitely yes	Probably no,	Definitely no	Definitely no
Chen et al. 2020 [15] China	Definitely yes	Definitely yes	Probably no,	Definitely no	Definitely no
Lei wu et al. 2015 China	Definitely yes	Definitely yes	Probably no,	Probably yes	Definitely no
Xia et al. 2021 China	Probably yes	Probably yes	Probably no	Probably yes	Probably no
Devkota et al. 2016 Nepal	Probably no	Probably yes	Probably no	Probably yes	Probably yes
Dhungana et a. 2022 [26] Nepal	Definitely yes	Definitely yes	Probably no,	Definitely no	Definitely no
Son et al. 2012 [27] Vietnam	Definitely yes	Definitely yes	Probably no,	Probably yes,	Probably yes,
De Souza et al. 2014 [16]	Probably yes	Probably yes	Probably no,	Probably yes,	Probably yes,

Supplementary Table S1. Quality appraisal of the included studies using the risk of bias instrument for Cross-Sectional Surveys Contributed by the CLARITY Group.

Author & Country	1. Is the source population representative of the population of interest?	2. Is the response rate adequate?	3. Is there little missing data?	4. Is the survey clinically sensible?	5. Is there any evidence for the reliability and validity of the survey instrument?
Brazil					
Lerner et al. 2013<br>Peru	Probably no	Probably yes	Probably no	Probably yes	Probably yes
Paquissi et al. 2016<br>Angola	Definitely no	Probably yes	Probably no	Probably yes	Definitely no
Okwuonu et al. 2014 [18]<br>Nigeria	Probably yes	Probably no	Probably yes	Probably yes,	Probably yes,
Iloh et al., 2013 [19]<br>Nigeria	Probably yes	Probably yes	Probably no,	Definitely yes (low risk of bias),	Probably yes,
Safro et al. 2018 [20]<br>Ghana	Definitely yes	Definitely yes	Probably no,	Probably yes,	Probably no
Harrison et al. 2021 [21]<br>Ghana	Probably yes	Probably yes	Probably no,	Probably yes,	Probably no
Gala et al. 2020 [23]<br>Botswana	Probably yes	Probably yes	Probably no,	Probably yes,	Probably no
Menanga et al. 2016 [24]<br>Cameroon	Probably yes	Probably yes	Probably no,	Probably yes,	Probably no
Duboz et al. 2014<br>Senegal	Probably yes	Probably yes	Probably no,	Probably yes,	Probably no
Zack et al. 2016<br>Tanzania	Definitely yes	Definitely yes	Probably no,	Probably yes,	Probably no
Nassr et al. 2019 [25]<br>Iraq	Probably yes	Probably yes	Probably no,	Definitely no	Definitely no
Zhang et al. 2019 [32]<br>Australia	Definitely yes	Probably yes	Probably no,	Probably yes,	Probably no

Supplementary Table S1. Quality appraisal of the included studies using the risk of bias instrument for Cross-Sectional Surveys Contributed by the CLARITY Group.

Author & Country	1. Is the source population representative of the population of interest?	2. Is the response rate adequate?	3. Is there little missing data?	4. Is the survey clinically sensible?	5. Is there any evidence for the reliability and validity of the survey instrument?
Liew et al. 2019 [38] Singapore	Definitely yes	Definitely yes	Probably no,	Probably yes,	Probably no
Tiffe et al. 2019 [35] Germany	Probably yes	Probably yes	Probably no,	Probably yes,	Probably no
Ham et al. 2011 [34] South Korea	Definitely yes	Probably yes	Probably no	Probably yes	Probably no
Cordero et al. 2011 [37] Spain	Definitely yes	Definitely yes (low risk of bias),	Probably no	Probably yes	Probably no
Sandoval et a. 2012 Chile	Definitely yes	Definitely yes	Probably no	Probably yes	Probably no
Ting li et al. 2016 Hong Kong	Definitely yes	Definitely yes	Probably no,	Probably yes,	Definitely yes
Murphy et al. 2015 Ireland	Definitely yes	Definitely yes	Probably no,	Probably yes,	Definitely no
Khayyat et al. 2017 Saudi Arabia	Probably no	Probably yes	Probably no	Probably yes	Probably no
Santosa et al. 2020<br><br>Sweden and China	Definitely yes	Probably yes	Probably no,	Probably yes,	Probably no

Note: for all questions, there are four choices of responses: Definitely yes (low risk of bias), Probably yes, Probably no, Definitely no (high risk of bias)
